# Effective-Component Compatibility of Bufei Yishen Formula III Combined with Electroacupuncture Suppresses Inflammatory Response in Rats with Chronic Obstructive Pulmonary Disease via Regulating SIRT1/NF-*κ*B Signaling

**DOI:** 10.1155/2022/3360771

**Published:** 2022-05-09

**Authors:** Fanli Jin, Lanxi Zhang, Kai Chen, Yufang Miao, Yang Liu, Yange Tian, Jiansheng Li

**Affiliations:** ^1^Henan Key Laboratory of Chinese Medicine for Respiratory Disease, Henan University of Chinese Medicine, Zhengzhou, Henan 450046, China; ^2^Collaborative Innovation Center for Chinese Medicine and Respiratory Diseases Co-Constructed by Henan Province & Education Ministry of P.R. China, Zhengzhou, Henan 450046, China; ^3^Department of Respiratory Diseases, The First Affiliated Hospital of Henan University of Chinese Medicine, Zhengzhou, Henan 450046, China

## Abstract

**Objective:**

To explore more efficient treatments for chronic obstructive pulmonary disease (COPD), effective-component compatibility of Bufei Yishen formula III (ECC-BYF III) and electroacupuncture were tested on rats with COPD, and silent information regulator transcript-1 (SIRT1)/nuclear factor-kappaB (NF-*κ*B) signaling was further investigated to interpret the therapy.

**Methods:**

In total, 70 rats were randomly divided into control (Control), model (Model), aminophylline (APL), ECC-BYF III, electroacupuncture (EA), ECC-BYF III+EA, and sham electroacupuncture (SA) groups. Cigarette smoke exposure combined with repeated bacterial infections was used to establish COPD models in 1–12 weeks. From 13 to 20 weeks, the ECC-BYF III and APL groups received corresponding drugs; the EA group received electroacupuncture therapy, wherein Dazhui (GV 14), Feishu (BL 13), and Shenshu (BL 23) points were selected; the ECC-BYF III+EA group received ECC-BYF III intragastrically combined with electroacupuncture; and the SA group received simulated electroacupuncture (nonacupoint). Pulmonary function, pulmonary histopathology, the expressions of SIRT1/NF-*κ*B signaling, and inflammation-related mRNA and protein were detected.

**Results:**

Significant deterioration was observed in pulmonary function and pulmonary histopathology in rats with COPD (*P* < 0.01), and inflammatory state was illustrated by increased levels of interleukin- (IL-) 6 and tumor necrosis factor alpha (TNF-*α*) and decreased levels of IL-10 (*P* < 0.01). After the intervention of APL, ECC-BYF III, EA, and ECC-BYF III+EA, both pulmonary function and pulmonary histopathology were improved (*P* < 0.05 and *P* < 0.01), whereas the levels of IL-6 and TNF-*α* were decreased and IL-10 was increased (*P* < 0.05 and *P* < 0.01). Additionally, the mRNA expressions of IL-6, TNF-*α*, NF-*κ*B, and acetylated NF-*κ*Bp65 (Ac-NF-*κ*B) were noted to decrease, and SIRT1 and IL-10 were increased (*P* < 0.05 and *P* < 0.01); the protein expression of SIRT1 was upregulated, and NF-*κ*Bp65 and Ac-NF-*κ*B were downregulated (*P* < 0.05 and *P* < 0.01). The effect of ECC-BYF III+EA was better in terms of improving pulmonary function and alleviating inflammation than that of the other treatment groups (*P* < 0.01 and *P* < 0.05).

**Conclusions:**

ECC-BYF III, electroacupuncture, and their combination can suppress inflammation, among which the combination therapy has been proven to be the most effective treatment, and the mechanism may be involved in activating SIRT1/NF-*κ*B signaling.

## 1. Introduction

Chronic obstructive pulmonary disease (COPD), characterized by persistent respiratory symptoms and airflow limitation, is a common, preventable, and treatable disease, which has been one of the leading causes of death and morbidity worldwide [1]. In a global context, the disability-adjusted life year of COPD was 2.6% and ranked eighth among the 315 Global Burden of Disease causes in 2015, affecting an estimated 174 million people in the same year [2]. Inflammation has been identified as one of the main pathogenesis of COPD, which refers to innate immunity and adaptive immunity [1, 3]. Pathogen-associated molecular patterns and damage-associated molecular patterns can recognize inhaled noxious particles and gases and can cause the release of proinflammatory cytokines [4–6]. This state could activate epithelial cells in the lungs and release inflammatory cytokines and chemokines, such as interleukin- (IL-) 6 and tumor necrosis factor alpha (TNF-*α*) [7, 8]. Macrophages further aggregate to release more proinflammatory IL-1-like cytokines, which, in turn, can lead to the persistent inflammation [3, 5, 9].

**Figure 1:**
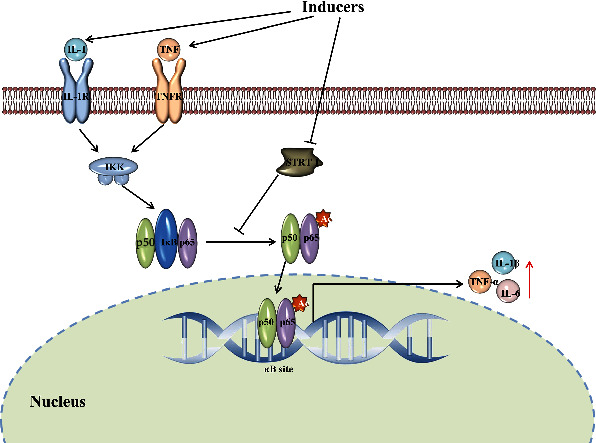
Schematic diagram of SIRT1/NF-*κ*B.

Silent information regulator transcript-1 (SIRT1) can deacetylate the subunit of nuclear factor-kappaB (NF-*κ*B), inhibit the binding of NF-*κ*B to the target gene, and reduce the expression of inflammatory cytokines [10]. When the cells were stimulated by inducers, such as free radicals and chemicals, the activity of SIRT1 was inhibited and the deacetylation of NF-*κ*Bp65 was reduced [11], thus regulating the release of inflammatory factors, such as promoting TNF-*α* and IL-1*β* and inhibiting the release of IL-10 (Figure 1) [12]. Therefore, increasing SIRT1 and inhibiting the activation of NF-*κ*B may play a potential role in the anti-inflammatory effect of COPD.

In recent years, traditional Chinese medicine (TCM) has made significant progress in treating COPD, including Chinese herbal formula, acupuncture, and acupoint sticking [13–16], among which Chinese herbal formula is the most widely used. The Bufei Yishen formula (BYF; pat. no., ZL.201110117578.1) has been known for its ability to improve the clinical symptoms of patients with COPD, including postponing the decline rate of pulmonary function, reducing the number of acute exacerbation, and improving the quality of life [17, 18]. Previous studies have shown that BYF can improve lung function and pathological injury of the lung tissue in rats with COPD, alleviate systemic and local inflammatory responses, and improve the protease-antiprotease imbalance and collagen deposition [14, 19–21]. BYF as a Chinese herbal formula is characterized by multicomponents, multipathways, and multitargets. Under the guidance of the theory of component compatibility [22], we screened the active components by systematic pharmacology; optimized the compatibility of the components by orthogonal design, total arrangement method, and activity evaluation; and further screened and optimized the components on the basis of animal and cell models [23, 24]. Finally, the effective-component compatibility of Bufei Yishen formula III (ECC-BYF III; patent application number, 201811115372.3) was obtained with clear ingredient, convenient dosage form, controllable quality, and the same curative effect as the BYF [25]. It is composed of icariin, astragaloside IV, nobiletin, ginsenoside Rh1, and paeonol.

Acupuncture, as one of the most popular treatments in alternative medicine and rehabilitation therapy, has a good clinical effect on COPD. The combination of acupuncture and conventional drug treatment can improve the pulmonary function of patients with COPD, increase partial pressure of oxygen, improve the patient's quality of life, enhance their respiratory muscle strength, improve exercise ability, and has high safety and few adverse events [26]. The remote effect of acupuncture stimulation can be achieved through the somatosensory-autonomic reflex. This reflex initially activates the peripheral sensory nerve fibers located in the dorsal root ganglion or trigeminal ganglion and then transmits the sensory information to the spinal cord and brain, thereby activating the peripheral autonomic nerve and realizing the regulation of various functions. The effects of limb area acupoint stimulation to inhibit the development of systemic inflammatory were mainly or partly related to activating efferent fibers of the vagus nerve, such as activating the vagus nerve-adrenal anti-inflammatory axis [27, 28].

Here, we use the theory of the TCM internal-external combined therapy theory as a guide, aimed to investigate the anti-inflammatory effect of ECC-BYF III, electroacupuncture (EA), and the combination of ECC-BYF III and EA in rats with COPD through the SIRT1/NF-*κ*B signaling.

## 2. Materials and Methods

### 2.1. Animals

The procedures of this study were approved by the Experimental Animal Care and Ethics Committees of the First Affiliated Hospital of Henan University of Chinese Medicine, and the ethical review approval number is YFYDW2019031. In total, 70 Sprague Dawley rats weighing 200 ± 20 g were used (Jinan Pengyue Experimental Animal Breeding Co., Ltd.; animal permit number, 1107261911000081).

### 2.2. Bacteria and Cigarettes

The bacteria (*Klebsiella pneumoniae*; strain ID, 46117) were purchased from the National Center for Medical Culture Collection. The bacteria were cultured in 10% solid nutrient agar (N8290, Solarbio), and it was adjusted to a bacterial solution of 6 × 10^8^ CFU/mL and intranasal drip (every 0.1 mL, once every 5 days) [29].

Hongqiqu® filter cigarettes (hard branch, flue-cured type; tar content, 10 mg; smoke carbon monoxide, 12 mg; smoke nicotine, 0.8 mg) were produced in Henan Zhongyan Industry Co., Ltd.

### 2.3. Drugs and Instrument

Chengdu Must Bio-Technology Co., Ltd. provided the compositions of the ECC-BYF III, which are icariin (MUST-17051810), astragaloside IV (MUST-17022804), nobiletin (MUST-16070901), 20-S-ginsenoside Rh1 (MUST-17030717), and paeonol (MUST-16071405). Aminophylline tablets were purchased from Xinhua Pharmaceutical Co., Ltd. (Shandong, China). Both ECC-BYF III and aminophylline were fully dissolved in 0.5% CMC-Na (sodium carboxymethyl cellulose; Hengxing Chemical Reagent Manufacturing Co., Ltd.; 500 g/bottle) and prepared into suspension before gavage. Acupuncture needles were purchased from Suzhou Acupuncture Supplies Co., Ltd. (Huanqiu Brand), and its specification is 0.30 mm × 13 mm, 100 pieces per box. The electroacupuncture apparatus was purchased from Suzhou Medical Supplies Factory Co., Ltd.

### 2.4. COPD Model Preparation and Administrations

In total, 70 rats were randomly divided into control (Control), model (Model), aminophylline (APL), ECC-BYF III, electroacupuncture (EA), ECC-BYF III+EA, and sham electroacupuncture (SA) groups.

Except for the rats of the Control group, COPD models were established in other rats. The cigarette smoke concentration was 3000 ± 500 ppm, the frequency was twice daily (40 min each time), and the bacterial solution was 6 × 10^8^ CFU/mL (0.1 mL, nasal drip, once every 5 days) [29]. Cigarette smoke combined with bacterial exposure was used to establish COPD models from 1 to 8 weeks, and cigarette smoke exposure alone was used from 9 to 12 weeks. For experimental rigor, the rats in the Control group received saline solution (0.1 mL, nasal drip, once every 5 days). At the 12^th^ week, 2 rats in each group were randomly selected for pulmonary function and pulmonary histopathology detection to determine whether the COPD model was successful or not.

**Figure 2:**
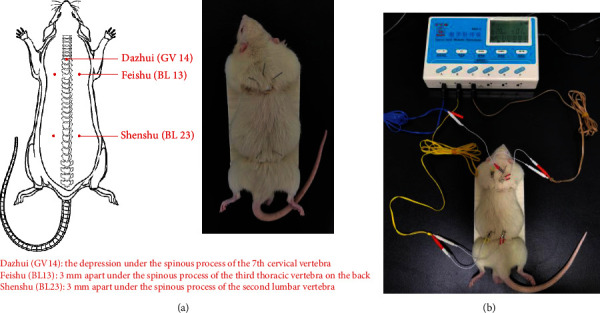
Rat acupoint diagram. (a) The location of Dazhui (GV 14), Feishu (BL 13), and Shenshu (BL 23) points [30]. (b) Schematic diagram of electroacupuncture treatment.

After completing the preparation of the COPD model, from 13 to 20 weeks, the rats in all groups received the corresponding treatments as follows: Control and Model groups, 0.5% intragastric CMC-Na (2 mL, 0.5 mL/100 g, bid); ECC-BYF III and ECC-BYF III+EA groups, ECC-BYF III intragastrically (5.5 mg/kg/day, 0.5 mL/100 g, bid); APL group, APL intragastrically (54 mg/kg/day, 0.5 mL/100 g, bid); and EA and ECC-BYF III+EA groups, electroacupuncture treatment three times a week (the operation method and acupoint locations [30] are shown in Figure 2). After being inserted, the stainless steel needles were connected with the electroacupuncture apparatus (alternating frequency, 1 Hz; intensity, 1 mA; time, 20 min, 3 times a week). The SA group received the same binding and electroacupuncture therapy, but the stimulation at point of nonacupoint.

### 2.5. Pulmonary Function

After the treatment at week 20, all rats were anesthetized intraperitoneally (1% Nembutal, 50 mg/kg). The forced vital capacity (FVC) and forced expiratory volume at 0.1 s (FEV0.1) were detected using FinePointe™ pulmonary function test system (Buxco, NY, USA). Additionally, FEV 0.1/FVC (%) was calculated.

### 2.6. Pulmonary Histopathology

The lung tissues were cut into 4 mm thick slices and stained with hematoxylin-eosin processing and observed using an optical microscope and photographic system (Olympus). The mean alveolar number (MAN) and mean linear intercept (MLI) of the lung slice were then calculated according to specific procedures and formulas [14]. Three major (c1/c2/c3) and 3 minor (d1/d2/d3) diameters of each bronchus passing through the center were measured, and the wall thickness (Wt) was calculated according to the following formula: Wt (*μ*m) = [(*c*1 − *d*1) + (*c*2 − *d*2) + (*c*3 − *d*3)]/(3 × 2). The outer tube wall area (*S*1), inner tube wall area (*S*2), and inner circumference (*C*) of the bronchus were measured, and the wall area (Wa) was calculated according to the following formula: Wa (*μ*m^2^) = (*S*1 − *S*2)/*C*.

### 2.7. Enzyme-Linked Immunosorbent Assay and Immunohistochemistry

Enzyme-linked immunosorbent assay (ELISA) was applied to detect the levels of IL-6, IL-10, and TNF-*α* in the lung tissues. The lung tissues were broken down to extract proteins, which are detected using the corresponding ELISA kit (BOSTER).

Immunohistochemistry (IHC) was applied to detect the protein expression levels of inflammatory factors in the lung tissues, which are IL-6, IL-10, and TNF-*α* (antibodies, ABclonal). Image-Pro Plus 6.0 (IPP 6.0) software (Media Cybernetics) measured the integral optical densities (IODs) of IL-10, IL-6, and TNF-*α*.

### 2.8. Quantitative Real-Time PCR and Western Blot

The quantitative real-time PCR (qRT-PCR) was applied to detect the mRNA expression levels of SIRT1, NF-*κ*Bp65, IL-6, IL-10, and TNF-*α* in the lung tissues. QIAzol® Lysis Reagent (QIAGEN) was used to extract the total RNA. HiScript® II Q RT SuperMix was used to synthesize reverse transcription. ChamQ Universal SYBR and specific primers (Table 1) were used to perform the reactions (Table 2) with the application of Biosystems 7500 instrument. Both HiScript® II Q RT SuperMix and ChamQ Universal SYBR were purchased from Vazyme Biotech Co., Ltd. GenScript Biotech Co., Ltd. provided the primers.

Western blot (WB) was applied to detect the protein expression levels of SIRT1, acetylated NF-*κ*Bp65 (Ac-NF-*κ*Bp65), and NF-*κ*Bp65 in the lung tissues, crus lung tissue and adding RIPA lysis to extract the total protein. Before detecting protein expression, the protein concentration was detected first using the BCA protein analysis kit (Solarbio). Next, protein denaturation, electrophoresis, and transfer to polyvinylidene fluoride were performed. Blocking fluid was used to block cross-reacting antibodies, and then, the blotted membranes were incubated with primary antibody and stored at 4°C. The following day, the secondary antibodies were incubated. Finally, the protein bands were visualized using the Super ECL Plus reagent (Solarbio).

### 2.9. Statistical Analysis

The data were processed using IBM SPSS Statistics for Windows, version 22.0. (IBM Corporation, Armonk), and the results were expressed as $\text{mean }\left(\overset{®}{x}\right)\pm \text{standard deviation }\left(s\right)$. The one-way ANOVA was used for comparison among groups. The least significant difference method was used for those who were consistent with the homogeneity of variance, whereas those who did not conform to the homogeneity of variance were analyzed using Dunnett's T3 method. The significance level was set as *P* < 0.05.

## 3. Results

### 3.1. Pulmonary Function

**Figure 3:**
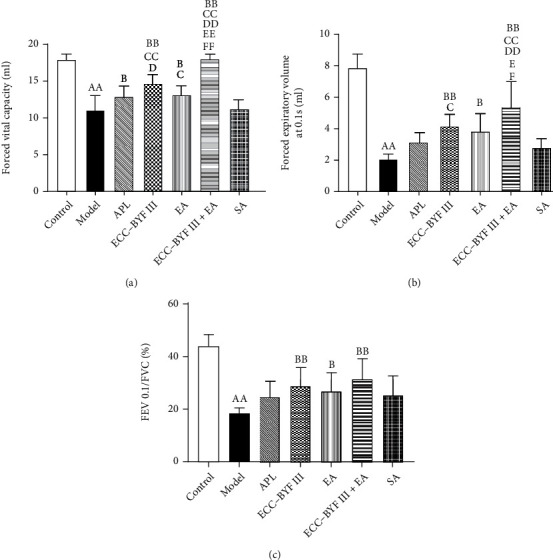
Changes in the pulmonary function in each group of rats at week 20: (a) FVC; (b) FEV 0.1; (c) FEV 0.1/FVC (%). Note: *n* = 6; ^a^*P* < 0.05 and ^aa^*P* < 0.01 vs. the Control group; ^b^*P* < 0.05 and ^bb^*P* < 0.01 vs. the Model group; ^c^*P* < 0.05 and ^cc^*P* < 0.01 vs. the SA group; ^d^*P* < 0.05 and ^dd^*P* < 0.01 vs. the APL group; ^e^*P* < 0.05 and ^ee^*P* < 0.01 vs. the ECC-BYF III group; ^f^*P* < 0.05 and ^ff^*P* < 0.01 vs. the EA group.

The diagnosis of COPD requires the assistance of pulmonary function tests, which is an important index for judging airflow limitation. As shown in Figure 3, FVC, FEV0.1, and FEV 0.1/FVC (%), as representative indexes of pulmonary function, have significantly decreased in rats in the model group (*P* < 0.01). After 8 weeks of treatment, ECC-BYF III, EA, and ECC-BYF III+EA showed improvements in FVC, FEV0.1, and FEV 0.1/FVC (%) (*P* < 0.05 and *P* < 0.01), whereas APL only improved FVC as compared with the Model group (*P* < 0.05). As compared with the rats in the SA group, ECC-BYF III, EA, and ECC-BYF III+EA showed improvement in the FVC (*P* < 0.05 and *P* < 0.01), whereas only ECC-BYF III and ECC-BYF III+EA showed improvement in the FEV0.1 (*P* < 0.05 and *P* < 0.01). The FVC of the rats in the ECC-BYF III and ECC-BYF III+EA groups and the FEV0.1 of the rats in the ECC-BYF III+EA group were observed to be better than those of the rats in the APL group (*P* < 0.05 and *P* < 0.01). The improvements of the FVC and FEV0.1 of the rats receiving ECC-BYF III+EA were better than those of the rats receiving ECC-BYF III and EA only (*P* < 0.05 and *P* < 0.01).

### 3.2. Pulmonary Histopathology

**Figure 4:**
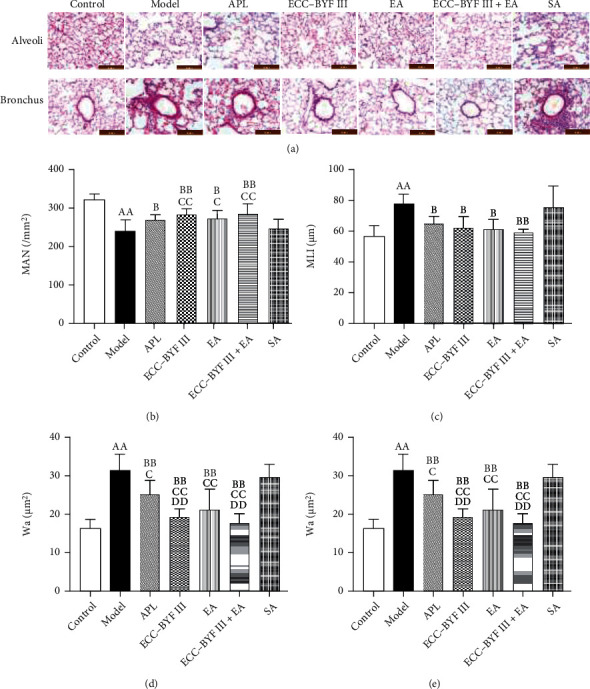
Changes in pulmonary histopathology in each group of rats: (a) pulmonary histopathology (hematoxylin-eosin, ×200); (b) MLI; (c) MAN; (d) Wt; (e) Wa. Note: *n* = 6; ^a^*P* < 0.05 and ^aa^*P* < 0.01 vs. the Control group; ^b^*P* < 0.05 and ^bb^*P* < 0.01 vs. the Model group; ^c^*P* < 0.05 and ^cc^*P* < 0.01 vs. the SA group; ^d^*P* < 0.05 and ^dd^*P* < 0.01 vs. the APL group.

Histopathological changes in the lung tissues in each group of rats were observed. As shown in Figure 4(a), it was observed that the structures of the bronchioles and alveoli of rats in the Control group were intact and there were fewer inflammatory cells; meanwhile, a great deal of inflammatory cells, serious alveolar dilatation, and alveolar wall rupture and fusion were observed in the Model and SA groups. After 8 weeks of treatment, the alveolar structure, inflammatory cells of the trachea, and alveolar surrounding in the lung tissues of treatment groups were reduced; in addition, the dilation of the alveolar cavity and the rupture and fusion of the alveolar wall were reduced.

As shown in Figures 4(b)–4(e), the MAN of the rats in the Model group decreased (*P* < 0.01), whereas the MLI, Wt, and Wa increased (*P* < 0.01). The treatment of ECC-BYF III, EA, ECC-BYF III+EA, and APL increased the MAN (*P* < 0.05 and *P* < 0.01) but decreased the MLI and Wa (*P* < 0.05 and *P* < 0.01) as compared with the Model group. However, Wt was only decreased in rats in the ECC-BYF III, EA, and ECC-BYF III+EA groups (*P* < 0.05 and *P* < 0.01). SA was not found to have a therapeutic effect; compared with it, ECC-BYF III, EA, and ECC-BYF III+EA increased the MAN (*P* < 0.05 and *P* < 0.01) and decreased Wt (*P* < 0.05 and *P* < 0.01); furthermore, ECC-BYF III, EA, ECC-BYF III+EA, and APL decreased Wa (*P* < 0.05 and *P* < 0.01). In addition, the decrease of Wa was noted to be more significant in ECC-BYF III and ECC-BYF III+EA than in APL (*P* < 0.01).

### 3.3. Inflammatory Factors

**Figure 5:**
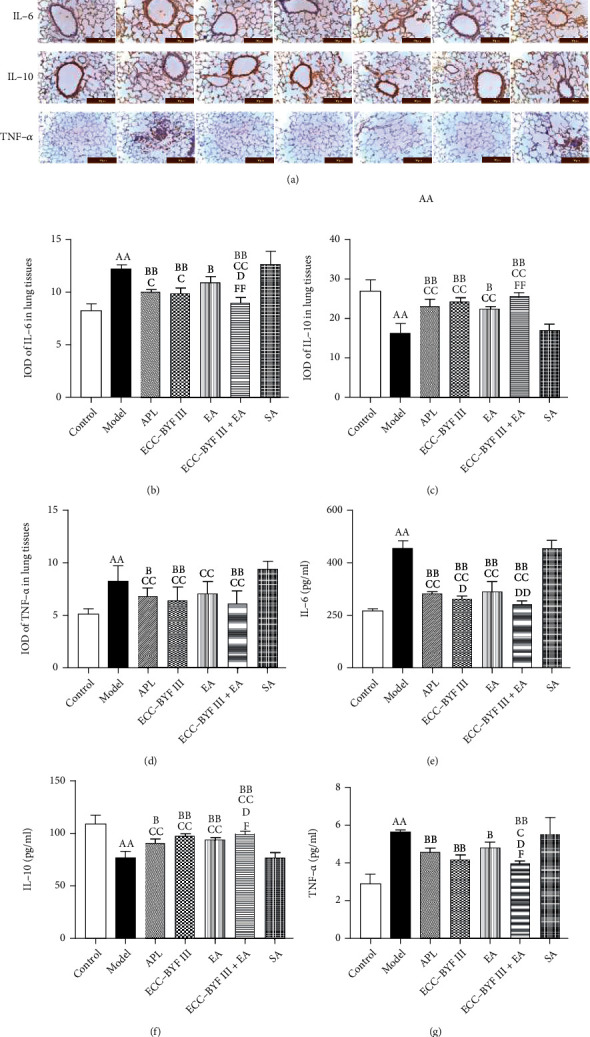
Changes in the inflammatory factor levels and protein expression. (a) IHC photograph. (b–d) IOD. (e–g) Levels of IL-6, IL-10, and TNF-*α* in the lung tissue. Note: *n* = 6; ^a^*P* < 0.05 and ^aa^*P* < 0.01 vs. the Control group; ^b^*P* < 0.05 and ^bb^*P* < 0.01 vs. the Model group; ^c^*P* < 0.05 and ^cc^*P* < 0.01 vs. the SA group; ^d^*P* < 0.05 and ^dd^*P* < 0.01 vs. the APL group; ^f^*P* < 0.05 and ^ff^*P* < 0.01 vs. the EA group.

ELISA and IHC were used to detect the levels and IODs of inflammatory factors (IL-6, IL-10, and TNF-*α*) in the lung tissues in each group of rats. As shown in Figure 5, the levels and IODs of IL-6 and TNF-*α* were increased (*P* < 0.01) and IL-10 was decreased (*P* < 0.01) in the Model group. ECC-BYF III, EA, ECC-BYF III+EA, and APL decreased the levels of IL-6 and TNF-*α* and the IODs of IL-6 (*P* < 0.05 and *P* < 0.01) and increased the levels and IODs of IL-10 (*P* < 0.05 and *P* < 0.01) as compared with the Model group, whereas ECC-BYF III, ECC-BYF III+EA, and APL decreased the IODs of TNF-*α* (*P* < 0.05 and *P* < 0.01). ECC-BYF III, EA, ECC-BYF III+EA, and APL decreased the levels of IL-6 and the IODs of TNF-*α* (*P* < 0.05 and *P* < 0.01) and increased the levels and IODs of IL-10 (*P* < 0.05 and *P* < 0.01) as compared with SA, whereas only ECC-BYF III+EA decreased the levels of TNF-*α* (*P* < 0.05) and ECC-BYF III, ECC-BYF III+EA, and APL decreased the IODs of IL-6 (*P* < 0.05 and *P* < 0.01). ECC-BYF III and ECC-BYF III+EA decreased the level of IL-6 (*P* < 0.05 and *P* < 0.01) as compared with APL, whereas only ECC-BYF III+EA decreased the IOD of IL-6 and the level of TNF-*α* (*P* < 0.05) and increased the level of IL-10 (*P* < 0.05). ECC-BYF III+EA decreased the IOD of IL-6 and the level of TNF-*α* as compared with EA (*P* < 0.05 and *P* < 0.01) and increased the level and IOD of IL-10 (*P* < 0.05 and *P* < 0.01).

### 3.4. mRNA Expressions of SIRT1, NF-*κ*B, IL-6, IL-10, and TNF-*α*

**Figure 6:**
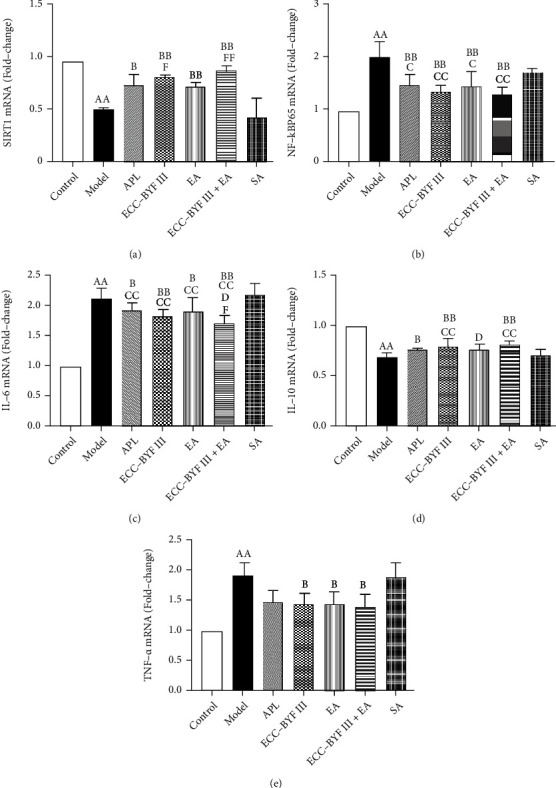
Changes of mRNA expressions of SIRT1, NF-*κ*Bp65, IL-6, IL-10, and TNF-*α*. Note: *n* = 6; ^a^*P* < 0.05 and ^aa^*P* < 0.01 vs. the Control group; ^b^*P* < 0.05 and ^bb^*P* < 0.01 vs. the Model group; ^c^*P* < 0.05 and ^cc^*P* < 0.01 vs. the SA group; ^d^*P* < 0.05 and ^dd^*P* < 0.01 vs. the APL group; ^f^*P* < 0.05 and ^ff^*P* < 0.01 vs. the EA group.

The mRNA expressions of SIRT1, NF-*κ*B, IL-6, IL-10, and TNF-*α* in the lung tissues of all rats were detected. As shown in Figures 6(a)–6(e), the mRNA expressions of NF-*κ*Bp65, IL-6, and TNF-*α* were observed to increase (*P* < 0.01) in the Model group, whereas SIRT1 and IL-10 decreased (*P* < 0.01). ECC-BYF III, EA, ECC-BYF III+EA, and APL decreased the mRNA expressions of NF-*κ*Bp65 and IL-6 (*P* < 0.05 and *P* < 0.01) and increased SIRT1 and IL-10 (*P* < 0.05 and *P* < 0.01) as compared with the Model group, whereas ECC-BYF III, EA, and ECC-BYF III+EA decreased TNF-*α* (*P* < 0.05). ECC-BYF III, EA, ECC-BYF III+EA, and APL decreased the mRNA expressions of NF-*κ*Bp65 and IL-6 (*P* < 0.05 and *P* < 0.01) as compared with SA; meanwhile, only ECC-BYF III and ECC-BYF III+EA increased IL-10 (*P* < 0.01). ECC-BYF III+EA decreased the mRNA expressions of IL-6 as compared with APL (*P* < 0.05). ECC-BYF III and ECC-BYF III+EA increased the mRNA expressions of SIRT1 (*P* < 0.05 and *P* < 0.01) and decreased IL-6 (*P* < 0.05) as compared with EA.

### 3.5. Protein Expressions of SIRT1, NF-*κ*B, and Ac-NF-*κ*Bp65

**Figure 7:**
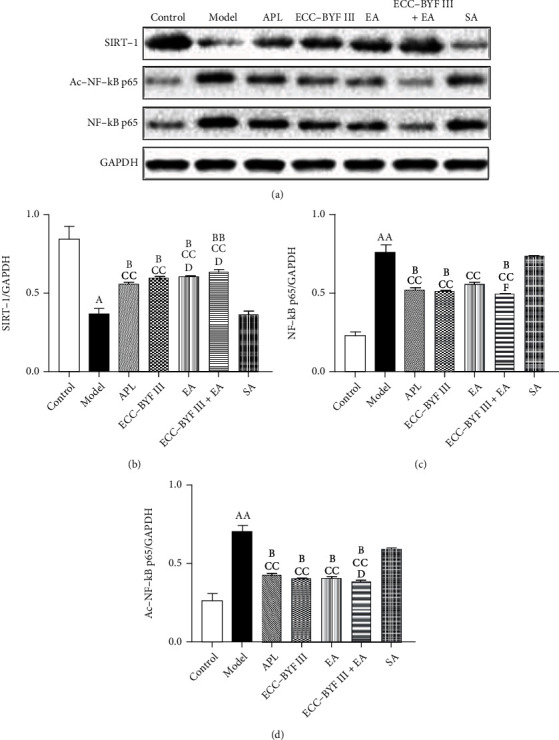
Changes of protein expressions of SIRT1, NF-*κ*Bp65, and Ac-NF-*κ*Bp65. Note: *n* = 3; ^a^*P* < 0.05 and ^aa^*P* < 0.01 vs. the Control group; ^b^*P* < 0.05 and ^bb^*P* < 0.01 vs. the Model group; ^c^*P* < 0.05 and ^cc^*P* < 0.01 vs. the SA group; ^d^*P* < 0.05 and ^dd^*P* < 0.01 vs. the APL group; ^f^*P* < 0.05 and ^ff^*P* < 0.01 vs. the EA group.

WB was used to detect the protein expressions of SIRT1/NF-*κ*B signaling-related protein (SIRT1, NF-*κ*B, and Ac-NF-*κ*B) in the lung tissues in all rats. As shown in Figures 7(a)–7(d), the protein expression of SIRT1 was decreased in the Model group (*P* < 0.05), whereas Ac-NF-*κ*Bp65 and NF-*κ*Bp65 were increased (*P* < 0.01). ECC-BYF III, EA, ECC-BYF III+EA, and APL increased the protein expression level of SIRT1 (*P* < 0.05 and *P* < 0.01) and decreased Ac-NF-*κ*Bp65 (*P* < 0.05) as compared with the Model group, whereas ECC-BYF III, ECC-BYF III+EA, and APL decreased NF-*κ*Bp65 (*P* < 0.05). ECC-BYF III, EA, ECC-BYF III+EA, and APL increased the protein expression level of SIRT1 (*P* < 0.01) and decreased Ac-NF-*κ*Bp65 and NF-*κ*Bp65 (*P* < 0.01) as compared with SA. EA and ECC-BYF III+EA increased the protein expression levels of SIRT1 (*P* < 0.05) as compared with APL, whereas only ECC-BYF III+EA decreased Ac-NF-*κ*Bp65 (*P* < 0.05). ECC-BYF III+EA decreased the protein expression levels of NF-*κ*Bp65 as compared with EA (*P* < 0.05).

## 4. Discussion

COPD has a long disease course and high morbidity and mortality rates; moreover, it is known to cause heavy economic burden. Inflammation, oxidative stress, and protease-antiprotease imbalance are considered to be related to the pathogenesis of COPD; among which inflammation plays a role in the whole pathogenesis and the persistent inflammation can cause irreversible airflow restriction or aggravate it [1]. The invasion of smoke and bacteria into the lung can activate inflammatory cells, such as monocytes/macrophages and neutrophils, and release inflammation-related mediators, which could then recruit and activate more inflammatory cells, resulting in the release of a large number of inflammatory factors and damage to the lung tissues [31]. Moreover, chronic irritants such as cigarette smoke can increase inflammation [1]. Chinese herbal formula and acupuncture belong to TCM, which are deemed to be safe and effective in the treatment of COPD [26, 32]. Molecular pharmacology results show that a variety of medicinal plants have shown remarkable anti-inflammatory effects, which play a role through traditional intervention methods. This effect leads to the increase of beneficial response of inflammatory diseases and the significant reduction of related complications and adverse effects [33]. Our previous studies have shown that BYF has a certain inhibitory effect on inflammation of COPD, which can be manifested as reduced inflammatory cell infiltration and expression of local and systemic inflammatory factors in the lung tissues [19, 20]. Acupuncture is one of the rehabilitation treatment methods of TCM, which is known for its high safety and few adverse events. It can effectively improve the pulmonary function, patients' quality of life, and exercise ability, and the treatment effect is more evident on the basis of the combination of conventional drugs [34]. Modern medicine has confirmed that the remote effect of acupuncture stimulation can be achieved through the somatosensory-autonomic reflex [27, 28]. Acupuncture can play a role by regulating the expression of genes and proteins and other biological processes [35, 36]. This study is aimed at investigating the anti-inflammatory effect of ECC-BYF III, EA, and the combination of ECC-BYF III and EA in rats with COPD.

Pulmonary function can accurately reflect the degree of airflow limitation and the severity of COPD, which FEV1/FVC is the gold standard. Thus, it can be a useful tool in diagnosing COPD and predicting disease progression [37]. In this study, the FEV0.1 and FVC was detected, and FEV0.1/FVC (%) was calculated to judge airflow limitation. This study shows that ECC-BYF III, electroacupuncture, and their combination showed significant effect in improving pulmonary function wherein that effect of the combined treatment is more evident. COPD is characterized by increased numbers of macrophages in the peripheral airways, lung parenchyma, and pulmonary vessels, together with increased activated neutrophils and lymphocytes [1]. We observed that there were severe pathological injuries of the lung tissue in the Model and SA groups; meanwhile, ECC-BYF III, electroacupuncture, and their combination showed improvement in varying degrees, among which the ECC-BYF III and the combination had the better effect.

Inflammation can directly cause lung damage, and it can also lead to mucus hypersecretion [29], protease-antiprotease imbalance, collagen deposition [38], and vascular remodeling [39, 40], which may accelerate the COPD process and increase the mortality of COPD. IL-6 is produced by macrophages, T lymphocytes, and other cells. Clinical detection of sputum, plasma, and bronchoalveolar lavage fluid of patients with COPD showed that the level of IL-6 is increased significantly [41, 42]. IL-10 is a protective cytokine, which is involved in the anti-inflammatory and inhibitory immune activity. The clearance of lung inflammation can be enhanced by reducing inflammatory cytokines secreted by neutrophils and airway alveolar macrophages in the lung, such as IL-1, IL-2, IL-8, and TNF-*α* [43]. TNF-*α* is a proinflammatory cytokine, which can be produced by various inflammatory cells. It can promote inflammatory cells to release other inflammatory cytokines, such as nitrogen groups and oxygen free radicals, and aggravate the inflammatory response [44]. The data of this study showed that the expression of proinflammatory cytokines (IL-6 and TNF-*α*) of rats with COPD was increased, whereas the anti-inflammatory cytokines (IL-10) were significantly decreased, suggesting that there was inflammatory reaction in COPD model rats. At week 20 (after 8 weeks of treatment), the level of IL-6 and TNF-*α* was decreased significantly in treatment groups, whereas IL-10 has increased significantly. Among them, ECC-BYF III+EA was both effective in reducing IL-6 and TNF-*α* and increasing IL-10, which shows better effectiveness than EA and APL, suggesting that ECC-BYF III, EA, and ECC-BYF III+EA could alleviate inflammation response in rats with COPD.

SIRT1/NF-*κ*B signaling-related molecules were detected to further reveal the potential mechanism of inflammation reduction in COPD. SIRT1 belongs to class III histone/protein deacetylases, which has anti-inflammatory, antiaging/senescence properties [45]. NF-*κ*B is one of the main substrates of SIRT1 and participates in physiological and pathological processes such as inflammation [46]. SIRT1 can deacetylate the subunit of NF-*κ*Bp65 and inhibit the binding of NF-*κ*B to target gene, thereby inhibiting the transcription of downstream inflammatory genes and the secretion of proinflammatory factors, such as IL-1*β*, IL-6, and TNF-*α* [10, 11]. SIRT1/NF-*κ*B signaling may have an effect on inhibiting inflammation of COPD [47]. As per the findings of this study, the level of NF-*κ*Bp65 and Ac-NF-*κ*Bp65 in the rats with COPD was significantly upregulated, whereas SIRT1 was significantly downregulated, suggesting that the activity of SIRT1 in rats with COPD was inhibited. However, the level of NF-*κ*Bp65 and Ac-NF-*κ*Bp65 was significantly downregulated after the intervention of ECC-BYF III, electroacupuncture, and their combination, whereas SIRT1 was significantly upregulated. The combination of ECC-BYF III and electroacupuncture was significantly superior to electroacupuncture in upregulating SIRT1, showing that it can upregulate SIRT1 and inhibit the acetylation of NF-*κ*B. Combined with the results of inflammatory factors, the combination of ECC-BYF III and electroacupuncture can inhibit inflammation by regulating SIRT1/NF-*κ*B signaling, thus playing an important role in the treatment of COPD.

To sum up, our study provides in *vivo* evidence that ECC-BYF III, electroacupuncture, and their combination can suppress inflammation, among which the combination therapy has been proven to be the most effective treatment, and the mechanism may be involved in activating SIRT1/NF-*κ*B signaling.

## Figures and Tables

**Table 1:** Primer sequences for qRT-PCR.

Gene	Product length	Primer	Primer sequence (5′-3′)
IL-6	83	Forward primer	GACTTCCAGCCAGTTGCCTT
		Reverse primer	AAGTCTCCTCTCCGGACTTGT
IL-10	147	Forward primer	CGCTGTCATCGATTTCTCCC
		Reverse primer	TGTCACGTAGGCTTCTATGC
TNF-*α*	88	Forward primer	CATCAAGAGCCCTTGCCCTA
		Reverse primer	CTGGAAGACTCCTCCCAGGTA
SIRT1	119	Forward primer	AGTAAGCGTCTTGACGGTAATCA
		Reverse primer	CTGCCACAGGAACTAGAGGAT
NF-*κ*Bp65	106	Forward primer	AGTCCCGCCCCTTCTAAAAC
		Reverse primer	CAATGGCCTCTGTGTAGCCC
GAPDH	72	Forward primer	ACGGGAAACCCATCACCATC
		Reverse primer	TACTCAGCACCAGCATCACC

**Table 2:** Reaction conditions of qRT-PCR.

Procedure	Temperature	Time	
Predenaturation	95°C	30 s	

Amplification curve	95°C	$\left.\begin{array}{c} 10\text{ s}\\ 30\text{ s} \end{array}\right\}$	40 circles
	60°C		

Dissolution curve	95°C	15 s	
	60°C	1 min	
	95°C	15 s	

## Data Availability

The datasets used and/or analyzed during the current study are available from the corresponding author on reasonable request.
